# Detrimental effects of hypercortisolism on brain structure and related risk factors

**DOI:** 10.1038/s41598-020-68166-0

**Published:** 2020-07-29

**Authors:** Yaxi Chen, Junhuai Zhang, Huiwen Tan, Jiaqi Li, Yerong Yu

**Affiliations:** 1 Department of Endocrinology and Metabolism, West China Hospital, Sichuan University, No. 37 Guoxue Xiang, Chengdu, Sichuan People’s Republic of China; 2 Department of Neurology, The People’s Hospital of Chongqing Yubei District, Chongqing, People’s Republic of China

**Keywords:** Endocrinology, Neurovascular disorders, White matter disease

## Abstract

Brain structural abnormalities are often observed on magnetic resonance imaging (MRI) scans of Cushing's syndrome patients, but the pathogenesis is not fully understood. To understand the relationship between brain structural abnormalities and potential risk factors in active Cushing's disease (CD) patients, a total of 101 treatment-naïve CD patients and 95 sex-, age- and education matched controls with non-functioning adenomas (NFA) underwent clinical evaluation and MRI investigation, and the relative risk factors were analyzed. 14 patients in sustained remission after transsphenoidal surgery were followed. Compared with the NFA subjects, the patients with CD had more cortical (*P* < 0.01) and subcortical atrophy (*P* < 0.01) and a higher prevalence of white matter hyperintensity (WMH) (*P* < 0.01). WMH severity in CD patients positively correlated with age (*r* = 0.532, *P* = 0.000), disease course (*r* = 0.257, *P* = 0.009), postprandial glucose (*r* = 0.278, *P* = 0.005), frequency of left ventricular hypertrophy (*r* = 0.398, *P* = 0.001) and hypothyroidism (*r* = 0.246, *P* = 0.014). The markers of cortical and subcortical atrophy (sylvian fissure ratio, bifrontal ratio, bicaudate ratio and third ventricle width) were positively associated with the progression of WMH in the CD patients. In the follow-up of 14 patients with CD, brain atrophy and WMH was partially reversible after correction of hypercortisolism. In conclusions, brain atrophy and WMH were more likely to appear in CD patients and were possibly partially reversible following correction of hypercortisolism.

Cushing's syndrome results directly from chronic exposure to excess glucocorticoids. Alongside various physical symptoms, patients with Cushing's syndrome, whether endogenous or exogenous, display a wide variety of neuropsychiatric and cognitive symptoms, which are indicative of the involvement of the central nervous system^1,2^. Brain structural abnormalities related to Cushing's syndrome have been repeatedly found, including smaller hippocampal volumes, enlarged ventricles, and cerebral atrophy^3^. Brain imaging studies and neuropsychological studies have indicated that hypercortisolism or supraphysiological levels of exogenous glucocorticoids are especially deleterious to the hippocampus and frontal lobes, and there were specific correlations between hippocampal volume and verbal learning, recall and memory function scores^4,5^. Although some of the deleterious effects of prolonged hypercortisolemia on cognitive functioning and brain volume are partly reversible after correction of hypercortisolism^6,7^, it remains unclear what pathophysiological processes are causing these reductions and reversibility, and we cannot draw conclusions concerning the underlying microstructural changes that are involved.

Patients with Cushing's syndrome has persistent cardiovascular risk factors^8^, which is closely related to cerebrovascular injury including white matter hyperintensities (WMH)^9^. Several recent studies examined white matter structural changes in Cushing's syndrome patients^10–12^, indicating that hypercortisolism affects the entire brain with white matter tracts demyelination^13^. Diastolic pressure and duration of hypertension was reported to positively correlated with white matter lesions severity in remitted patients with Cushing's syndrome^14^. It is imperative to uncover the underlying pathways through which hypercortisolism leads to structural changes, so effective medical treatment and preventive strategies of cardiovascular risk factors for the detrimental effects of hypercortisolism might be implemented.

In this study, we report the gray matter and white matter structural abnormalities found by MRI images in 101 patients with active Cushing’s disease and the reversibility of these structural changes in 14 subjects who had been in remission of hypercortisolism for at least one year after surgery. The risk factors related to brain structural abnormalities in Cushing's syndrome were also analyzed.

## Results

### Characteristics of patients with Cushing's disease and age- and sex-matched NFA subjects

The clinical characteristics of the patients and the NFA group are presented in Table 1. There were no significant differences between subjects with CD and the NFA with respect to age, sex, and education. The patients with CD had higher waist circumferences, body mass index (BMI), levels of total cholesterol (TC), triglyceride (TG), HbA1C, and fasting blood glucose, and systolic blood pressure (SBP) and diastolic blood pressure (DBP) than the NFA group. The results of 24-h dynamic electrocardiogram showed that 14 CD patients had arrythmia including sinus tachycardia, sinus bradycardia, myocardial ischemia and premature ventricular beat. The ultrasonic cardiogram examinations showed that 55 CD patients had left ventricular hypertrophy (LVH). Of the 101 CD participants (11% with macroadenomas and 89% with microadenomas), 67.5% had received lipid-lowering drugs, 45.5% and 15.8% were diagnosed with diabetes and prediabetes, respectively, 77.2% were diagnosed with hypertension, and 39.6% were diagnosed with hypokalemia.

**Table 1:** Participant characteristics.

Characteristics	CD	NFA	*P* value
Number of subjects (male/female)	101 (17/84)	95 (19/76)	0.567
Macroadenomas (n)	10	0	
Microadenomas (n)	91	95	
Mean age (year)	37.4 ± 12.6	35.4 ± 11.5	0.215
Years of education	12.2 ± 5.7	13.1 ± 6.6	0.683
Education level			0.848
Low (n/%)	44 (43.5%)	40 (42.1%)	
Medium (n/%)	41 (40.6%)	37 (38.9%)	
High (n/%)	16 (15.8%)	18 (18.9%)	
Smoking history (n)	3 (2.9%)	2 (2.1%)	
Drinking history (n)	6 (5.9%)	4 (4.2%)	
Disease course (year)	3.9 ± 3.4	–	
Waist circumference (cm)	91.8 ± 10.1	80.2 ± 8.6	< 0.01
BMI (kg/m^2^)	25.0 ± 3.5	21.6 ± 2.7	< 0.01
Total cholesterol (mmol/l)	5.4 ± 1.2	4.3 ± 1.1	< 0.05
Triglycerides (mmol/l)	1.6 ± 1.0	1.4 ± 0.7	< 0.05
HbA1c (%)	6.3 ± 1.2	5.6 ± 0.4	< 0.05
Fasting serum glucose (mmol/l)	5.9 ± 2.1	5.1 ± 1.8	0.117
Systolic blood pressure (mm/Hg)	147.0 ± 21.7	114 ± 16.5	< 0.01
Diastolic blood pressure (mm/Hg)	97.5 ± 15.3	82 ± 6.9	< 0.01
Pre-diabetes (%)	15.8%	2.1%	< 0.01
Diabetes (%)	45.5%	4.2%	< 0.01
Hypertension (%)	77.2%	2.1%	< 0.01
Hypothyroidism (%)	26.7%	0	
Hypogonadism (%)	36.6%	0	

Anterior pituitary hormone function was normal in the NFA subjects, while in the 101 CD patients, 26.7% had central hypothyroidism and 36.6% had central hypogonadism.

### Brain atrophy assessment in CD patients and NFA subjects

**Figure 1:**
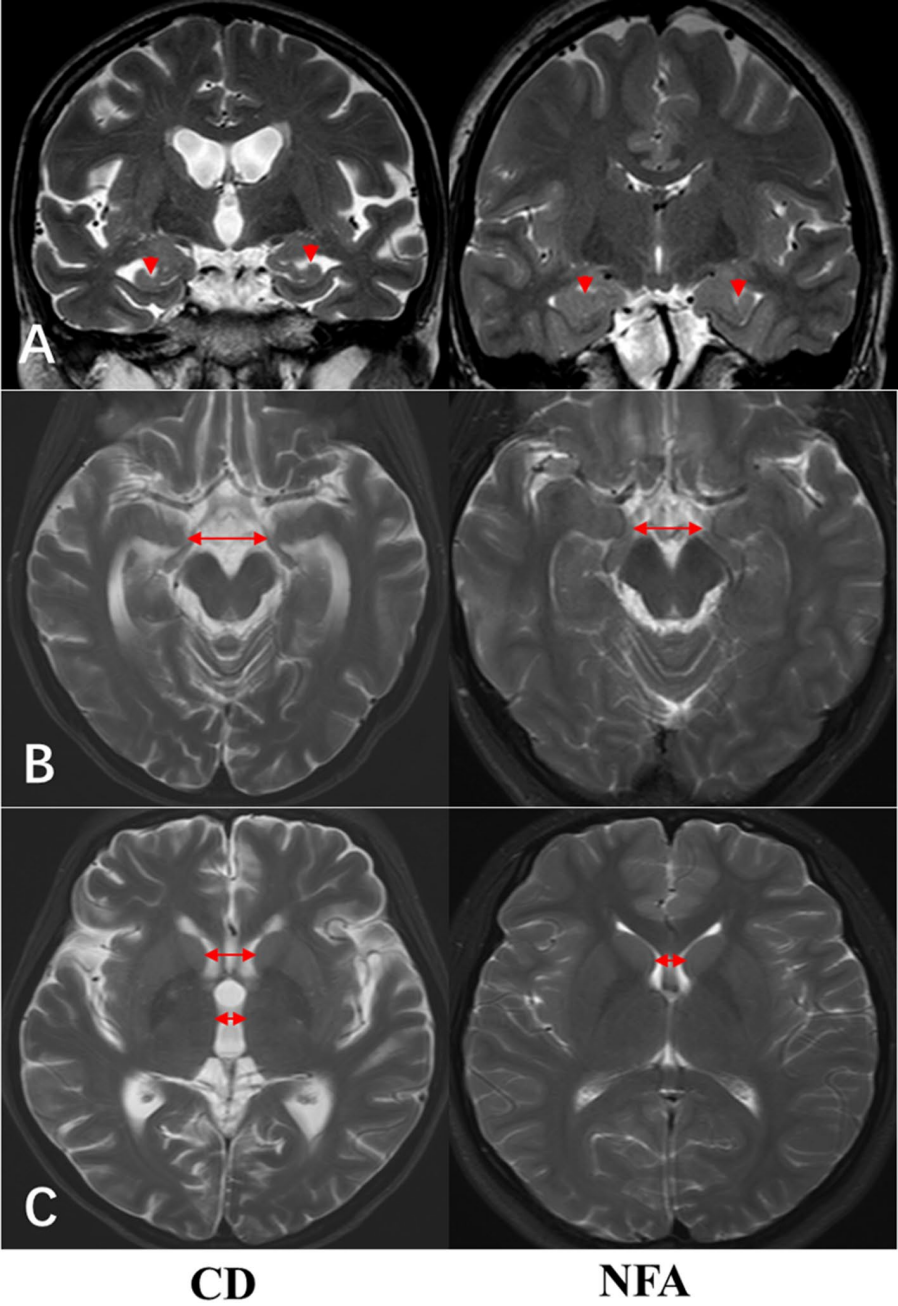
(**A**) MRI Image of a 43-year-old female CD patient with hippocampal atrophy compared with NFA subject; (**B**) MRI Image of a 45-year-old female CD patient with temporal lobe atrophy compared with NFA subject; (**C**) MRI Image of a 40-year-old female CD patient with subcortical atrophy compared with NFA subject.

The results of the cerebral atrophy rating scale assessment before and after adjusting for SBP and BMI are shown in Table 2. Compared with NFA after adjustment, the subjects in the CD group showed decreased hippocampal height (*P* < 0.01) and increased temporal horn width (*P* < 0.01), suggesting temporal lobe atrophy in the CD patients (Fig. 1A, B). Increasing sylvian fissure ratio (SFR) (*P* < 0.01) and frontal interhemispheric fissure ratio (FFR) (*P* < 0.01) indicated cortical atrophy, while increasing bicaudate ratio (BCR) (*P* = 0.012) indicated subcortical atrophy in the CD patients, accompanied by widened third ventricle (*P* < 0.01) (Fig. 1C). Furthermore, the patients with CD tended to have more lacunar infarcts than NFA subjects (8/101 vs. 2/95, *P* = 0.055).

**Table 2:** Cerebral atrophy by linear measurement.

	CD (n = 101)	NFA (n = 95)	*P* value
**Before adjusting for SBP and BMI**			
Hippocampal height (mm)	7.01 ± 1.12	9.21 ± 0.98	< 0.01
Width of temporal horn (mm)	3.63 ± 1.22	2.63 ± 0.77	< 0.01
Uncotemporal index	0.204 ± 0.061	0.191 ± 0.021	0.040
SFR	0.033 ± 0.009	0.022 ± 0.020	< 0.01
FFR	0.029 ± 0.008	0.019 ± 0.005	< 0.01
BFR	0.304 ± 0.024	0.293 ± 0.019	< 0.01
BCR	0.094 ± 0.019	0.080 ± 0.014	< 0.01
Third ventricle width	4.78 ± 1.58	3.48 ± 1.19	< 0.01

	CD (n = 32)	NFA (n = 32)	*P* value
**After adjusting for SBP and BMI**			
Hippocampal height (mm)	6.93 ± 1.04	9.16 ± 0.95	< 0.01
Width of temporal horn (mm)	3.95 ± 1.55	2.51 ± 0.79	< 0.01
Uncotemporal index	0.217 ± 0.103	0.192 ± 0.022	0.184
SFR	0.033 ± 0.009	0.022 ± 0.004	< 0.01
FFR	0.039 ± 0.016	0.021 ± 0.011	< 0.01
BFR	0.301 ± 0.030	0.296 ± 0.021	0.401
BCR	0.092 ± 0.022	0.080 ± 0.014	0.012
Third ventricle width	4.84 ± 1.510	3.740 ± 1.305	< 0.01

### White matter lesions in the CD patients and NFA subjects by Scheltens scale

**Figure 2:**
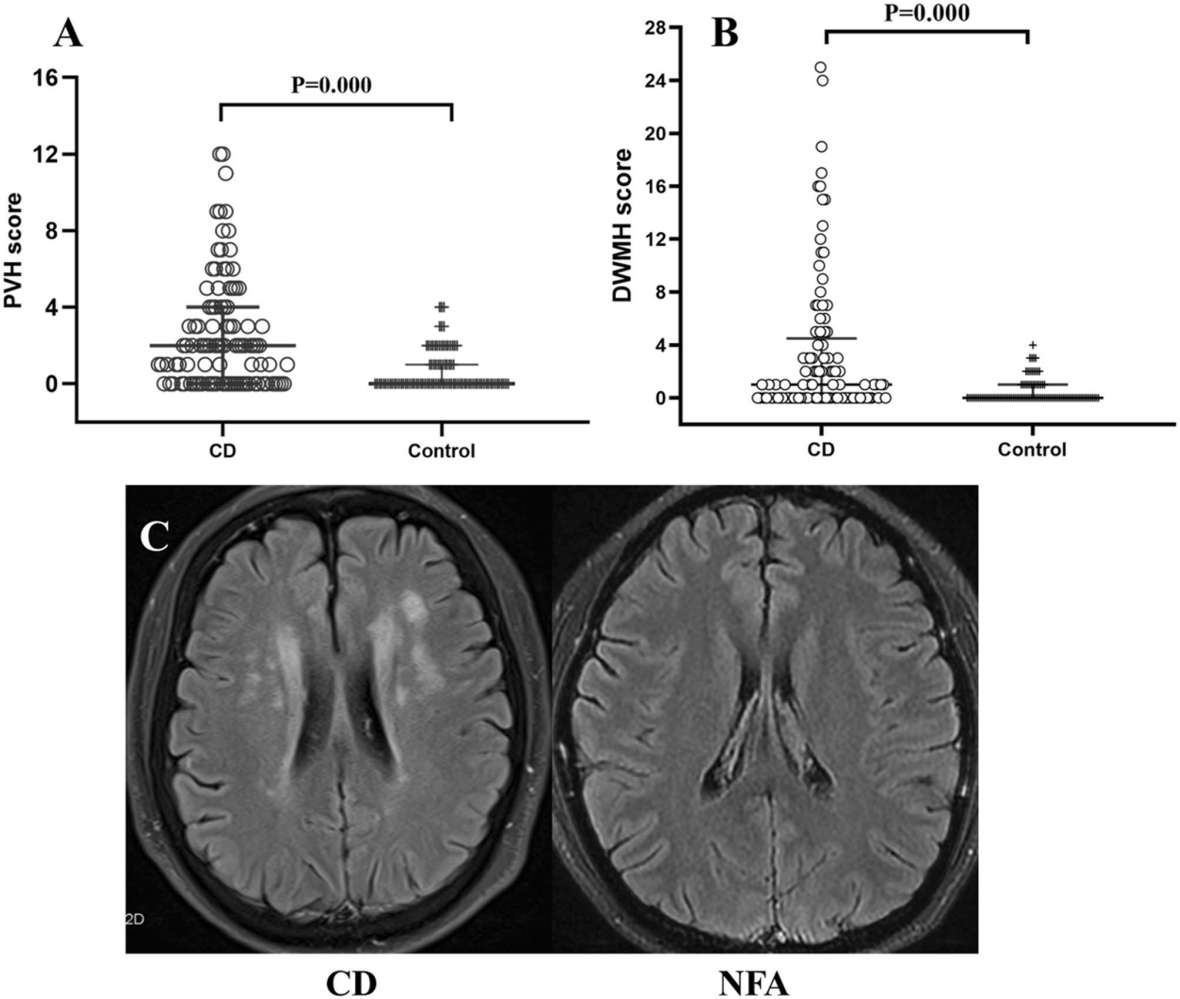
(**A**) and (**B**) MRI findings in NFA subjects and CD patients for PVH (Scheltens scale 0–12) and DWMH (Scheltens scale: 0–36). Lines represents median with interquartile range. (**C**) FLAIR image of a CD patient with severe WMH compared with NFA subject.

White matter lesions were common in the CD patients. We used Scheltens rating to evaluate the WMH (Fig. 2A,B). The prevalence of WMH was 68% in the CD patients and 27% in the NFA subjects. The deep white matter hyperintensity (DWMH) scores and periventricular hyperintensity (PVH) scores were obviously higher in the CD group than in the NFA group. In the periventricular region, the CD group had a higher likelihood of receiving a score of 2 (4) than a score of 0 (1) in the NFA group (*P* < 0.001). In the deep white matter region, the frontal lobe (*P* < 0.001), parietal lobe (*P* < 0.001), occipital lobe (*P* < 0.001), temporal lobe (*P* < 0.001) and basal ganglia (*P* < 0.001) showed higher Scheltens scores in the CD subjects than in the NFA group, while there was no difference in the infratentorial area between the two groups (*P* = 0.051). Figure 2C shows a representative FLAIR image of the PVH and DWMH in a CD patient and a NFA subject.

### Analysis of risk factors for WMH severity in CD patients by Fazekas scale

We used the Fazekas classification method to divide the CD patients into 3 groups according to the WMH grade by univariate analyses (Table 3) and correlation analysis (with significant difference, Table 4). The CD patients with moderate to severe WMH were older and had a longer disease course, higher blood glucose and TG levels, higher frequency of diabetes, LVH and arrhythmia than the patients in the other categories. WMH severity in CD patients positively correlated with age (*r* = 0.532, *P* = 0.000), disease course (*r* = 0.257, *P* = 0.009), fasting blood glucose(*r* = 0.212, *P* = 0.033), postprandial glucose (*r* = 0.278, *P* = 0.005), frequency of diabetes (*r* = 0.245, *P* = 0.013), LVH (*r* = 0.398, *P* = 0.001) and hypothyroidism (*r* = 0.246, *P* = 0.014).

**Table 3:** Characteristics in subgroups based on WMH severity in CD patients.

	Grade 0 (n = 35)	Grade 1 (n = 40)	Grade 2 (n = 26)
**Clinical characteristic**s			
Gender(female/male)	27/6	35/7	23/3
Age (years)	29.4 ± 9.1	38.6 ± 10.9**	46.61 ± 12.58##**
Disease course (years)	2.97 ± 2.58	4.06 ± 3.06	5.27 ± 4.54*
Smoking history (n)	1 (3.03%)	0 (0)	2 (8.69%)
Drinking history (n)	1 (3.03%)	3 (7.14%)	2 (7.69)
BMI (kg/m^2^)	24.98 ± 4.26	24.63 ± 3.04	25.62 ± 3.19
Waist circumstance (cm)	90.05 ± 11.54	91.92 ± 8.89	93.73 ± 10.06
SBP (mmHg)	142.3 ± 21.2	150.4 ± 24.3	148.5 ± 17.5
DBP (mmHg)	96.7 ± 14.1	100.8 ± 18.4	94.1 ± 10.7
Hypertension (%)	66.67%	83.33%	80.76%
Fasting blood glucose (mmol/L)	5.10 ± 0.92	6.19 ± 2.37*	6.39 ± 2.68*
2 h-postprandial glucose (mmol/L)	8.39 ± 4.89	9.68 ± 6.14	12.24 ± 4.84**
Diabetes (%)	27.27%	54.76%*	57.69%*
TG (mmol/L)	1.36 ± 0.78	1.52 ± 0.78	2.02 ± 1.51*
TC (mmol/L)	5.37 ± 1.30	5.20 ± 1.22	5.64 ± 1.21
LDL-C (mmol/L)	3.16 ± 1.11	2.98 ± 1.03	3.33 ± 1.03
Arrythmia (%)	9.1%	16.67%	19.23%**
LVH (%)	25%	66.67%**	77.27%**
**Anterior pituitary function**			
GH (ng/ml)	0.28 (1.53)	0.29 (0.76)	0.15 (0.22)
TSH (mIU/L)	1.23 (2.26)	1.10 (1.59)	1.01 (1.52)
FT4 (pmol/L)	13.67 ± 2.76	12.74 ± 2.63	11.60 ± 3.77*
FT3 (pmol/L)	3.49 ± 0.83	3.14 ± 0.65	3.03 ± 0.76
PRL (ng/ml)	13.99 (24.25)	22.58 (34.88)	18.80 (28.1)
ACTH (ng/L)	60.9 (110.0)	74.09 (110.35)	67.0 (98.6)
PTC-8 am (nmol/L)	834.2 ± 254.1	926.4 ± 305.3	836.3 ± 281.2
PTC-0 am (nmol/L)	640 (781.4)	638 (917.6)	626.3 (912.2)
UFC (ug/24 h)	737.3 (1634.2)	911.4 (1,382.8)	872.0 (1,195.0)
Hypothyroidism (%)	14.3%	27.5%	42.3% *
Hypogonadism (%)	31.4%	42.8%	35.3%
**MRI findings**			
Hippocampal height (mm)	7.11 ± 1.07	6.97 ± 1.03	6.94 ± 1.34
Width of temporal horn (mm)	3.45 ± 0.95	3.81 ± 1.26	3.60 ± 1.49
SFR	0.0313 ± 0.0086	0.0317 ± 0.0085	0.0369 ± 0.0108#*
FFR	0.0278 ± 0.0071	0.0277 ± 0.0132	0.0317 ± 0.0180
Uncotemporal index	0.2106 ± 0.0987	0.2006 ± 0.0263	0.2023 ± 0.0290
BFR	0.2954 ± 0.0194	0.3072 ± 0.0241*	0.3086 ± 0.0257*
BCR	0.0862 ± 0.0153	0.0965 ± 0.0187*	0.0994 ± 0.0229**
Third ventricle width (mm)	4.32 ± 1.22	4.71 ± 1.48	5.48 ± 1.90**#

**Table 4:** Correlation analysis of WMH severity and characteristics in CD patients.

	Coefficient of correlation	*P* value
Age (years)	0.532	0.000
Disease course (year)	0.257	0.009
Fasting blood glucose (mmol/L)	0.212	0.033
2 h-postprandial glucose (mmol/L)	0.278	0.005
Diabetes (%)	0.245	0.013
LVH (%)	0.398	0.001
FT4 (pmol/L)	− 0.292	0.006
Hypothyroidism (%)	0.246	0.014
SFR	0.197	0.049
BFR	0.200	0.044
BCR	0.241	0.015
Third ventricle width (mm)	0.242	0.015

The markers of cortical and subcortical atrophy: SFR (*r* = 0.197, *P* = 0.049), BFR (*r* = 0.200, *P* = 0.044), BCR (*r* = 0.278, *P* = 0.005) and third ventricle width (*r* = 0.242, *P* = 0.015) were positively associated with the progression of WMH in the CD patients.

### White matter lesions in patients in remission of CD by Scheltens scale

Of the 196 participants, 14 CD (14 female/0 male, 35.6 ± 14.1 years old) patients who were in remission after surgery and 14 NFA subjects (14 female/0 male, 35.7 ± 13.9 years old) were routinely followed. There were no differences between the groups in age, sex and educational level. At baseline, the CD patients had decreased hippocampal height (*P* = 0.000), increased temporal width (*P* = 0.003), SFR (*P* = 0.000), FFR (*P* = 0.000), BCR (*P* = 0.038), BFR (*P* = 0.004), and third ventricle width (*P* = 0.000), and higher DWMH (*P* = 0.025) and PVH (*P* = 0.037) scores than the NFA subjects.

**Figure 3:**
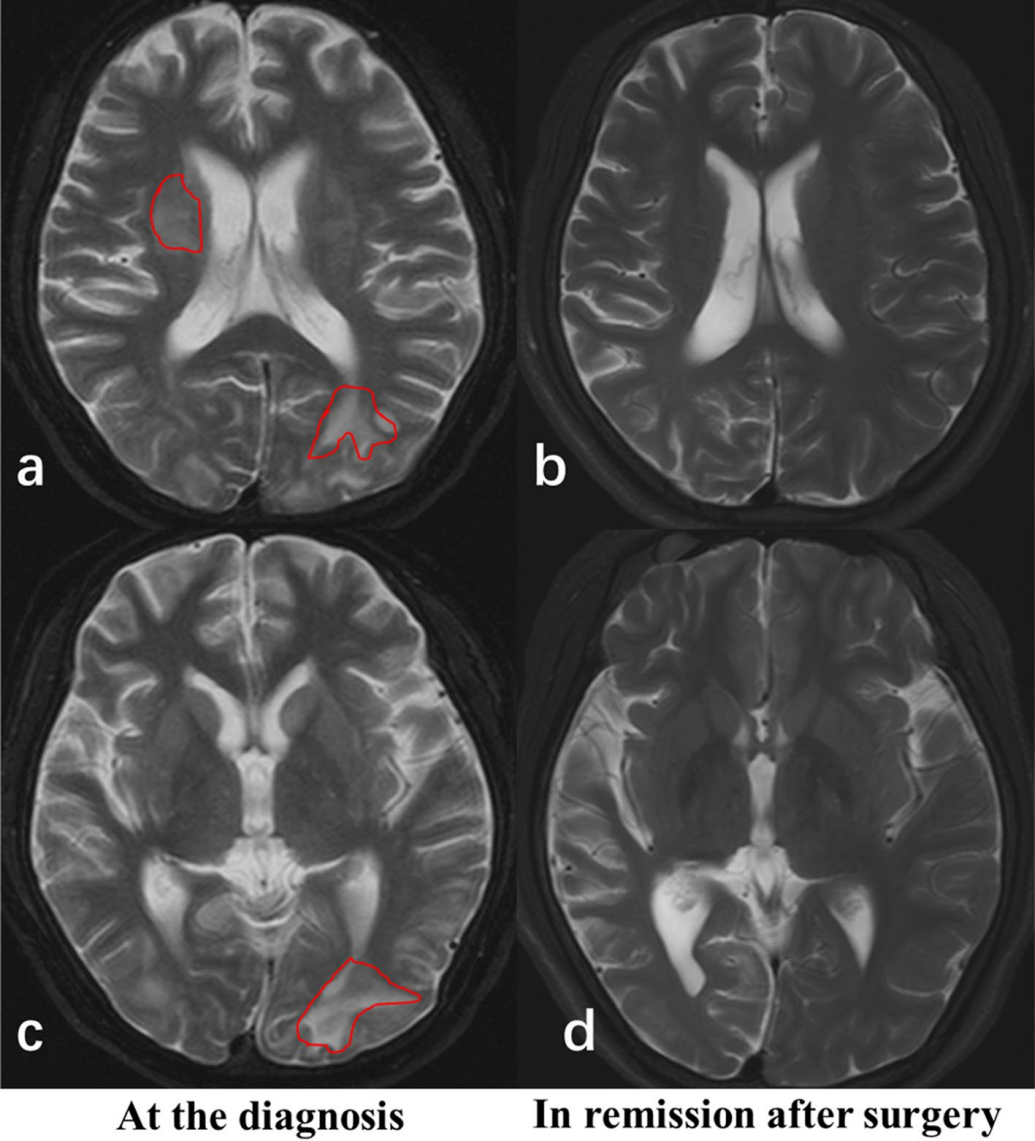
MRI image of a 19-year-old CD patient at the diagnosis (**a**, **c**) and in remission after surgery (**b**, **d**).

During the 25.4 ± 10.7 months after surgery, all 14 CD patients showed biochemical evidence of remission with normal morning cortisol suppression (23.35 ± 9.40 nmol/L). 8 CD patients experienced improvement in WMH scoring, while the rest 6 patients had no significant changes. The remitted CD patients experienced increased hippocampal height (*P* = 0.047), BCR (*P* = 0.024) and third ventricle width (*P* = 0.002), suggesting an improvement in brain atrophy. In addition, the PVH (*P* = 0.017) and DWMH scores, especially in the parietal lobe (*P* = 0.027) and temporal lobe (*P* = 0.042), substantially decreased compared with baseline (Table 5, Fig. 3).

**Table 5:** Neuroradiological structure and WMH scores (Scheltens scale) of CD prior to treatment (baseline), after surgical remission and sex- and age-matched NFA subjects.

	NFA (n = 14)	CD baseline (n = 14)	CDin remission (n = 14)	*P* value CD baseline versus NFA	*P* value CD baseline versus in remission
**Neuroradiological structure**					
Hippocampal height (mm)	8.61 ± 0.74	6.52 ± 0.92	6.98 ± 0.59	0.000	0.047
Width of temporal horn (mm)	2.41 ± 0.61	3.32 ± 0.87	3.13 ± 0.54	0.003	0.502
Uncotemporal index	0.1885 ± 0.0187	0.1962 ± 0.0219	0.2064 ± 0.0133	0.329	0.173
SFR	0.0184 ± 0.0039	0.0331 ± 0.0084	0.0320 ± 0.0151	0.000	0.736
FFR	0.0259 ± 0.0117	0.0321 ± 0.0089	0.0287 ± 0.0094	0.000	0.406
BFR	0.2851 ± 0.0125	0.3067 ± 0.0212	0.3052 ± 0.0198	0.004	0.678
BCR	0.0756 ± 0.0148	0.0889 ± 0.0173	0.0817 ± 0.0103	0.038	0.024
Third ventricle width (mm)	3.11 ± 0.81	4.95 ± 1.31	4.21 ± 1.09	0.000	0.002
**WMH score (Scheltens scale)**					
Frontal lobe	0 (0)	0 (2.25)	0 (1.25)	0.058	0.257
Parietal lobe	0 (1)	0 (4)	0 (1)	0.034	0.027
Temporal lobe	0 (0)	0 (3.25)	0 (0.25)	0.042	0.042
Occipital lobe	0 (1)	0 (1.75)	0 (1)	0.168	0.084
Basal ganglia	0 (1)	0 (1.5)	0 (1)	0.121	0.461
Infratentorial region	0 (0)	0 (0)	0 (0)	0.180	0.180
DWMH	0.5 (2)	0.5 (16)	0 (6)	0.025	0.048
PVH	0 (2)	1 (7.25)	0 (3)	0.037	0.017

## Discussion

### Hypercortisolemia causes brain atrophy changes

This study demonstrated that brain structural abnormalities are common in patients with active CD. We detected temporal lobe atrophy, cortical atrophy, subcortical atrophy and a widened third ventricle in CD patients when compared with age- and sex-matched controls. These detrimental effects of chronic glucocorticoid excess have been described in previous clinical and experimental studies^3,15^. The data obtained from 14 CD patients in remission with a mean follow-up of 25.4 ± 10.7 months showed that hippocampal height, BCR and third ventricle width increased after the correction of hypercortisolemia, suggesting an improvement in brain atrophy. Our results supported the suggestion that chronic glucocorticoid excess causes brain atrophy changes.

Some clinical studies have confirmed that the structural abnormalities revealed by MRI in patients with active Cushing's syndrome might be related to psychological morbidity and cognitive impairment^16^, and following the successful treatment of hypercortisolism, both the physical features and the psychiatric symptoms tended to substantially improve^17,18^. The neurotoxic effects of corticosteroid excess on the CNS have been well recognized in experimental animal studies^19,20^. To develop an effective medical treatment for these harmful effects of hypercortisolism on brain structure and psychological/cognitive impairment, it is important to gain more insight into these pathological processes.

### White matter hyperintensities indicate that hypercortisolemia affects the entire brain

Widespread white matter hyperintensities throughout the brain in the CD patients was another finding in this study. Although the average age was only 37.4 years old in our CD patients, the prevalence of WMH was 67%, much higher than that in NFA (27%) and healthy community-based populations^21^. WMH is a characteristic of white matter injury. We found that the DWMH scores and PVH scores were obviously higher in the CD group. Predictably, we observed that the markers of cortical atrophy and subcortical atrophy were closely associated with the progression of WMH in CD patients. These white matter lesions improved in 14 patients, especially in the parietal lobe and temporal lobe, following the successful treatment of hypercortisolism. These findings are in line with the recent studies which examined white matter structural changes in patients with Cushing's syndrome, suggesting hypercortisolism affects the entire brain with indications of demyelination of the white matter tracts^12,13^. However, in a cross-sectional study, Werff reported that CD patients with long-term remission showed widespread reduction of white matter integrity in the brain, suggesting persistent structural effects of hypercortisolism^10^. In a latest research, the alterations of white matter in 35 CD patients seem to be persist after remission^22^, which is in contrast with our results. The difference may due to the limitation of our small sample size. More research with sufficient cases is needed to clarify how hypercortisolism affect white matter tissue injury.

### Hypothyroidism may be involved in brain structural changes in CD patients

The functions of the pituitary-thyroid axis are suppressed in patients with Cushing’s syndrome because of a direct effect of cortisol on TSH secretion^23^. Hypothyroidism is a recognized cardiovascular risk factor, and reduced cardiac output and tissue perfusion as well as decreased tissue oxygen utilization are common in patients with hypothyroidism. Additionally, cognitive dysfunction is a common feature of hypothyroidism, and there have been reports that hypothyroidism may be a risk factor for Alzheimer’s disease^24,25^. In this study, we found that the incidence of central hypothyroidism in the patients with WMH was at least double that of the patients without WMH, and the FT_4_ levels were much lower in the CD patients with moderate or severe WMH than in those without WMH. It is logical to speculate that hypothyroidism may be involved in brain structural changes in CD patients.

### Cerebral small vessel disease might be one of the potential pathophysiological links between hypercortisolism and brain structural abnormalities

It is known that WMH, indicating white matter tract demyelination, is an imaging manifestation of denervation of nerve conduction fibers caused by diffuse cerebral ischemia and belongs to a type of cerebral small vessel disease (CSVD)^26^. Many studies have confirmed that CSVD is a key pathological link in the early stages of many brain diseases^27^. Given that WMH and brain atrophy are characteristic imaging changes in CSVD, we hypothesize that brain structural abnormalities on MRI images of patients with Cushing's syndrome may be related to CSVD. We analyzed the relationship between a variety of traditional cardiovascular risk factors and WMH in CD patients, and the results showed that age and fasting and postprandial blood glucose and TG levels tended to rise in accordance with WMH grade. These well-established factors were as applicable to CD patients as they were to the general population. In particular, we found a higher prevalence of LVH and arrhythmia with WMH progression, raising the hypothesis that excess cortisol-induced cerebral ischemia changes may share the same well-established mechanism of cerebrovascular endothelial dysfunction and inflammatory response^28^.

Vascular conditions can, at least partially, contribute to brain structural abnormalities in CD patients. Not only absolute blood pressure levels, but even their fluctuations over time can have detrimental effects on brain parenchyma^29^ and are associated with cognitive dysfunction^30^. Additionally, cardio-vascular risk factors in CD patients, including but not limited to hypertension and diabetes mellitus, can significantly impair cerebral hemodynamics, which is a contributor to neurocognitive dysfunction and is also amenable to reverse^31^. The relation between WMH and diabetes is still debated, a recent study found that WMH volume and number of WMH lesions were significantly associated with diabetes^32^. It is still not clear whether WMH is related to hypercortisolism regardless of diabetes in our research due to lack of evidenced analysis.

### Mineralocorticoid receptor (MR) overactivation may also play a role in brain structural abnormalities in Cushing's syndrome

Glucocorticoids, for instance cortisol, usually bind with glucocorticoid receptors (GRs) and mineralocorticoid receptors (MRs). 11β-HSD2 enzyme converts cortisol into inactive cortisone (which can’t bind with MR) to protect from the MRs effect. However, elevated cortisol levels overcome the ‘protective’ role of 11β-HSD2, leading to MR excess and successive multiple effects^33^. The incidences of hypertension and hypokalemia in our CD patients were 77.2% and 39.6%, respectively, suggesting MR hyperactivation at the renal level. Moreover, the incidences of arrythmia and LVH in our CD patients were 13.8% and 54.4%, respectively, suggesting MR hyperactivation at the cardiovascular level^34^.

It is worth noting that 11β-HSD2 is not expressed in the hippocampus or other limbic structures^35^; therefore, hypercortisolism increases the occupation of MRs/GRs. Studies on hippocampal cell cultures showed that supraphysiological doses of glucocorticoids lead to a reversible phase of atrophy of the apical dendrites of pyramidal neurons^36^. Accumulating evidence has indicated an important role for the activation of MRs in the pathophysiology of vascular damage in the heart and kidney^37^. Given that MRs are highly expressed in many brain tissues^38^ and cerebral vessels^39^, we speculated that MR overactivation may also play a role in the brain structural abnormalities in Cushing's syndrome. In a latest animal research, we observed that MR and GR overactivation can cause brain atrophy and vascular apoptosis in a cortisol-excess animal model^40^. Further exploration of the cerebral molecular mechanisms underlying the detrimental effects of Cushing’s disease could help prevent nervous system complications and cardiovascular disorders.

Our study has some limitations. First, our sample size was too small to allow sufficiently powered statistical such as ordinal logistic regression to be performed. The number of patients in the comparative analysis of clinical characteristics between subgroups based on WMH was unbalanced and small. Further examination with larger numbers of CD patients is needed to confirm our findings. Moreover, due to the retrospective design, no measures were used to assess psychological morbidity or cognitive impairment and we could not use more advanced MRI techniques to measure brain volume, such as voxel-based morphometry in which 3D sequence is needed.

## Conclusion

Brain atrophy and white matter hyperintensities are characteristic manifestations of brain MRI images in Cushing's syndrome. The pathogenesis of the brain structural abnormalities induced by chronic glucocorticoid excess appears to be multifactorial and is not yet fully understood. In addition to glucocorticoid excess itself, cerebral small vessel disease might be one potential pathophysiological link between long-term hypercortisolemia and brain structural abnormalities. High blood pressure, elevated blood glucose, associated hypothyroidism and MR overactivity may all be involved in this pathological process. To develop an effective medical treatment for the detrimental effects of glucocorticoid excess, it is imperative to understand the underlying pathways through which hypercortisolemia leads to brain structural changes.

### Patients and methods

#### Participants

One hundred and one consecutive patients who had undergone evaluation and treatment for Cushing's disease (CD) at West China Hospital between 2013 and 2019 were involved in this study. We retrospectively reviewed our data. The study was approved by the Ethics Committee of West China Hospital and conformed to the tenets of the Declaration of Helsinki. Written informed consent was obtained from all subjects in our study.

All 101 CD patients were diagnosed based on agreed guidelines^41^, with clinical manifestations and positive biochemical tests. The mean age of the patients at the time of diagnosis was 37.4 ± 12.6 years. Eighty-four patients were female (83%). Ninety-five sex-, age- and education matched treatment-naïve subjects diagnosed with nonsecreting pituitary microadenoma (NFA) were recruited in West China hospital as controls.

Of these 101 CD patients, 14 subjects (all females) who were in sustained remission after transsphenoidal surgery were followed for one to four years (the median follow-up period was 25.4 ± 10.7 months). Biochemical remission after surgery was defined as suppression of plasma cortisol to less than 50 nmol/l after 1 mg overnight dexamethasone and a normal urinary free cortisol (UFC) excretion^8^. 14 sex-, age- and education matched treatment-naïve subjects diagnosed with nonsecreting pituitary microadenoma (NFA) were recruited in West China hospital as controls. No subjects had a history of stroke, long-term physical illness, or other neuropsychiatric diseases. Only right-handed subjects were included.

#### Clinical interview and biochemistry

All subjects underwent a complete clinical interview, physical examinations and blood biochemical tests, including electrolytes. Clinical variables related to cardiovascular risk, including age, smoking history, drinking history, systolic blood pressure (SBP), diastolic blood pressure (DBP), hemoglobin A1c (HbA1C), fasting and 2-h postprandial plasma glucose, and lipid profiles were collected. All CD patients received 24-h dynamic electrocardiogram and ultrasonic cardiogram examinations. Hypertension was defined as a blood pressure greater than or equal to 140/90 mmHg^32^. Prediabetes was defined by a fasting glucose level between 5.6 mmol/L and 6.9 mmol/L or a 2-h postprandial load glucose level between 7.8–11.0 mmol/L. Diabetes was determined as a fasting glucose level ≥ 7.0 mmol/L or 2-h postprandial load glucose levels ≥ 11.1 mmol/L, according to the World Health Organization (WHO) diagnostic criteria for diabetes^42^.

In addition to plasma/urine cortisol and ACTH level measurements, dynamic endocrine tests (including dexamethasone suppression tests, desmopressin (DDAVP) stimulation tests) and in some cases inferior petrosal sinus sampling required for establishing CD diagnosis, the following pituitary-target gland hormones were also tested in all participants: thyroid-stimulating hormone (TSH), free thyroxine (FT_4_), luteinizing hormone (LH), follicle-stimulating hormone (FSH), estrogen (E_2_), testosterone (T), growth hormone (GH) and prolactin (PRL). Fourteen patients who were in remission and had follow-up after surgery were reevaluated at 6-month to 12-month intervals, and the endocrine evaluation consisted of measurement of serum cortisol after 1 mg overnight dexamethasone and 24-h UFC excretion along with assessment of anterior pituitary hormone function.

### MRI scans

All subjects were examined with a 3-T Signa MRI scanner (GE, DISCOVERY MR750). A line connecting the anterior commissure and posterior commissure (AC-PC) was drawn on the midsagittal slice and used for orientation of the remaining series. T1-weighted, T2-weighted, and FLAIR axial sections (repetition/echo/inversion time (TR/TE/TI): 9,000/95/2,475 ms, matrix 256 × 256) were obtained through the entire brain, parallel to the AC-PC line, with 5-mm contiguous slices. Custom graphics software was locally developed by using Win10 Windows. The slices containing regions of interest were first identified on digital images. Corresponding cuts were then displayed on the graphics workstation and interactively outlined with a mouse-controlled cursor. The MRI images were annually reexamined and reviewed in 14 CD patients with persistent remission after surgery every 6 months on the same scanner.

### Cerebral atrophy rating scale

To assess cerebral atrophy, we used linear measurements^43^. We adopted the bicaudate ratio (BCR) and bifrontal ratio (BFR)^44^ as measures of internal cerebral atrophy, the sylvian fissure ratio (SFR) and frontal interhemispheric fissure ratio (FFR)^44^ as measures of external cerebral atrophy, the uncotemporal index^45^, and hippocampal formation height and width of the temporal horn as a measure of temporal atrophy^46^. Detailed descriptions of the linear measurements above are listed in Table 6. Furthermore, a lesion was considered a lacunar infarct if its score was hypointense on T1 and FLAIR images and if its appearance was unlike a perivascular space.

**Table 6:** Cerebral atrophy rating scale.

**Internal cerebral atrophy**	
BCR	Minimum distance between frontal horns divided by the distance between the inner tables of the skull along the same line
BFR	Distance between the tips of the frontal horns divided by the distance between the inner tables of the skull along the same line
**External cerebral atrophy**	
SFR	Average of the maximum width of the two sylvian fissures on the section showing them at their widest, divided by the trans-pineal coronal inner table diameter
FFR	Maximal width of the interhemispheric fissure divided by the trans-pineal coronal inner table diameter
**Temporal atrophy**	
Uncotemporal index	Ratio of distance between the unci of temporal lobes to the distance between outer margins of temporal lobes
Hippocampal formation height	Measured on a plane parallel to the brainstem axis plane where the hippocampal formation was highest
Width of the temporal horn	Measured on the same plane used for hippocampal height measurement

### White matter hyperintensities by Scheltens rating

WMH were considered present if these were hyperintense on FLAIR images and not hypointense on T1WI^47^. To measure WMH in the cross-sectional (part 1) and longitudinal design (part 3) in different subregion, we used the modified Scheltens rating scale WMH from two different areas, including periventricular hyperintensity (PVH) and deep white matter hyperintensity (DWMH), were rated in a semiquantitative way^48^. The total PVH score was added with a range of 0–6, and DWMH was calculated with a range of 0–36 (Table 7). All MRI scans were independently rated by two raters blinded to the disease status of the participants. In the case of disagreements, consensus readings were held. The interrater reliability coefficient was 0.96–0.98 for the linear measures, and a paired t-test revealed no significant differences between the means obtained by the two raters.

**Table 7:** White matter hyperintensities by Scheltens rating.

**PVH (range 0–6)**	
Adjacent to the lateral ventricles	0-none 1-lesion ≤ 5 mm 2-lesions > 5 mm
Adjacent to the frontal and occipital horns	
**DWMH (range 0–36)**	
Frontal region	0 (none) 1 (lesions ≤ 3 mm, n ≤ 5) 2 (lesions ≤ 3 mm, n ≥ 6) 3 (4 mm ≤ lesions ≤ 10 mm, n ≤ 5) 4 (4 mm ≤ lesions ≤ 10 mm, n ≥ 6) 5 (lesions ≥ 11 mm, n ≥ 1) and 6 (large confluent lesions)
Parietal region	
Occipital region	
Temporal region	
Basal ganglia	
Infratentorial region	

### White matter hyperintensities by Fazekas rating

In part 2, the severity of WMH was rated with the Fazekas scale and divided into a non-WMH group, a mild-WMH group, and a moderate-severe WMH group^46^. The Fazekas scale provides evaluations from PVH and DWMH. PVH was graded as 0 = absence, 1 = “caps” or pencil-thin lining, 2 = smooth “halo,” and 3 = irregular PVH extending into the deep white matter. DWMH were rated as 0 = absence, 1 = punctate foci, 2 = beginning confluence of foci, and 3 = large confluent areas. The sum of the PVH and DWMH scores provides a total WMH score, and the participants were then divided into a non-WMH group (total score = 0), a mild-WMH group (1 ≤ total score ≤ 2), and a moderate-severe WMH group (total score ≥ 2). Vascular risk factors and radiological features were compared among the groups.

### Statistical analysis

Quantitative data are expressed as the mean ± standard deviation (SD) or as the median with interquartile range (IQR) when the data showed a nonnormal distribution. The normal distribution of the between-group parameters was examined using the Kolmogorov–Smirnov test. Values were compared between groups using Student’s *t*-test for means, the chi-square test for proportions and the Mann–Whitney U test for nonparametric data. Propensity score matching was used to compare linear measurement between groups. Correlations were assessed using the Spearman’s test for non-parametric variables. Values of *P* < 0.05 were regarded as significant. All statistical analyses were performed using the software SPSS version 21.

### Ethical approval

Our study was approved by the Ethics Committee of West China Hospital. Written informed consent was obtained from all subjects in our study.
